# Age Effects on Cognitive and Physiological Parameters in Familial Caregivers of Alzheimer's Disease Patients

**DOI:** 10.1371/journal.pone.0162619

**Published:** 2016-10-05

**Authors:** Márcio Silveira Corrêa, Bruno Lima Giacobbo, Kelem Vedovelli, Daiane Borba de Lima, Pamela Ferrari, Irani Iracema de Lima Argimon, Julio Cesar Walz, Elke Bromberg

**Affiliations:** 1 Laboratório de Biologia e Desenvolvimento do Sistema Nervoso, Faculdade de Biociências, Pontifícia Universidade Católica do Rio Grande do Sul, Porto Alegre, RS, Brazil; 2 Programa de Pós-Graduação em Biologia Celular e Molecular, Pontifícia Universidade Católica do Rio Grande do Sul, Porto Alegre, RS, Brazil; 3 Instituto de Geriatria e Gerontologia, Pontifícia Universidade Católica do Rio Grande do Sul, Porto Alegre, RS, Brazil; 4 Laboratório de Psiquiatria Molecular, Hospital de Clínicas de Porto Alegre, Porto Alegre, RS, Brazil; 5 Instituto Nacional Ciência e Tecnologia—Medicina Translacional (INCT-TM), RS, Brazil; 6 Faculdade Unilasalle, Canoas, RS, Brazil; Hokkaido Daigaku, JAPAN

## Abstract

**Objectives:**

Older familial caregivers of Alzheimer’s disease patients are subjected to stress-related cognitive and psychophysiological dysfunctions that may affect their quality of life and ability to provide care. Younger caregivers have never been properly evaluated. We hypothesized that they would show qualitatively similar cognitive and psychophysiological alterations to those of older caregivers.

**Method:**

The cognitive measures of 17 young (31–58 years) and 18 old (63–84 years) caregivers and of 17 young (37–57 years) and 18 old (62–84 years) non-caregiver controls were evaluated together with their salivary cortisol and dehydroepiandrosterone (DHEA) levels, as measured by radioimmunoassays and ELISA assays of brain-derived neurotrophic factor (BDNF) in serum.

**Results:**

Although younger caregivers had milder impairments in memory and executive functions than older caregivers, their performances fell to the same or lower levels as those of the healthy older controls. Decreases in DHEA and BDNF levels were correlated with the cognitive dysfunctions observed in the older and younger caregivers, respectively. Cortisol at 10PM increased in both caregiver groups.

**Discussion:**

Younger caregivers were prone to cognitive impairments similar to older caregivers, although the degree and the neuropsychological correlates of the cognitive dysfunctions were somewhat different between the two groups. This work has implications for caregiver and care-recipient health and for research on the neurobiology of stress-related cognitive dysfunctions.

## 1 Introduction

In the next decades, a huge increase in dementia cases is expected as a consequence of global population aging [1]. Among dementias, Alzheimer’s disease (AD) is the most prevalent [2]. Assistance to AD patients is primarily provided by familial caregivers, mainly spouses and daughters [3,4], who are faced with an overwhelming and challenging task of providing care that can last a long period of time. Due to the high dependence level of their demented relatives, caregivers are often subjected to an assistance-related physical and emotional burden [5].

Until now, studies on familial caregivers have mainly focused on the effects of emotional distress on older subjects, such as the patients’ spouses [6]. The chronic stress suffered by these caregivers, in association with their advanced age, predisposes them to psychological, behavioral and physiological risk factors for cognitive decline and dementia [3,7,8]. Studies have indicated impairments in executive functions (working memory, attention and processing speed) and declarative memory of older caregivers. These cognitive domains depend heavily on two of the most sensitive brain structures to stress, the prefrontal cortex (PFC) and the hippocampus [9], which contain a particularly high density of cortisol receptors [10], a hormone that is expected to be altered in acute and chronic stress [11].

Most studies of cortisol levels (measured in blood or saliva samples) in dementia caregivers suggest an up-regulation of the hypothalamic-pituitary-adrenal (HPA) axis and, as a consequence, hypercortisolemia [12,13]. Moreover, aging can also predispose humans to elevated cortisol levels [14,15]. The negative effects of hypercortisolemia on neuronal plasticity, survival and neurogenesis are well documented [16,17] and could be related, at least partly, to the cognitive impairment observed in caregivers [18; 19]. However, cortisol is not the only steroid that undergoes altered levels in response to stress. As shown in former studies [20], stressful events also increase dehydroepiandrosterone (DHEA) levels, which have anti-glucocorticoid effects and lessen the negative effects of cortisol on the central nervous system [21]. Thus, it is suggested that the cortisol/DHEA ratio is a more reliable marker for cognitive changes than cortisol or DHEA alone [22]. In fact, a previous study has shown that increased cortisol/DHEA ratios are related to the cognitive decline observed in dementia caregivers [19].

The evidence discussed above also suggests that cortisol and DHEA alterations are not the only responsible factors for the cognitive impairments observed in caregivers. Recently, our research group showed a decrease in brain-derived neurotrophic factor (BDNF) levels in familial caregivers [19]. Animal [23] and human [24] studies have shown that peripheral and central BDNF are correlated. This neurotrophin, which modulates synaptic plasticity, neurogenesis and neuronal survival [25], is reduced in chronic stress situations [26] and is expected to decrease with age [27], although controversy exists regarding this issue [28,29]. There is also evidence that serum BDNF levels correlate with cognitive performance in different physiological and clinical conditions [24,25,30]. However, its participation in the cognitive impairments of dementia caregivers has not yet been proven.

Until now, no study has specifically evaluated the cognitive performance of younger caregivers, such as patients’ children. However, this is an important issue because, as caregivers, they are also prone to mood alterations (i.e., depression or anxiety), poorer sleep quality, social isolation and less time to take care of themselves, among other negative effects related to their caregiver activity [8,31]. Thus, younger caregivers are exposed to almost the same risk factors of cognitive decline as older caregivers (with the evident exception of age). Therefore, the aim of this study was to compare the effects of chronic stress related to caregiving activities on the cognition of younger and older caregivers and to investigate physiological parameters that may be modulated by stress and related to cognitive performance. Our hypotheses were that (I) cognitive impairment would be observed in both age groups, being more pronounced in the older caregivers; (II) hormonal and BDNF levels would be altered in the caregivers, with greater stress effects on older caregivers, and (III) hormonal and BDNF levels would be significantly related with the cognitive performance of caregivers.

## 2 Material and Methods

### 2.1 Participants

Seventeen younger (48.82 ± 2.07 years; 15 women) and seventeen older (74.16 ± 1.82 years; 15 women) family caregivers of AD patients were recruited from the Brazilian Alzheimer Association (Porto Alegre, Brazil). Additionally, seventeen younger (46.23 ± 1.37 years, 14 women) and eighteen older (68.22 ± 1.51 years, 13 women) control (non-caregiver) subjects were recruited from the community. Exclusion criteria were visual or hearing impairments, the use of medications that could interfere with the HPA axis or cognition, past or current use of psychoactive drugs, unstable medical conditions, neurological trauma or diseases, scores on Mini Mental State Exam (MMSE) [32] compatible with dementia (cut off < 26) and scores on Beck Depression Inventory (BDI) and Beck Anxiety Inventory (BAI) indicative of severe depressive (cut off > 30) or anxiety (cut off > 30) symptoms [33]. Psychological and physical stress symptoms were evaluated according to the Lipp Inventory of Stress Symptoms for Adults (ISSL) [34,35], a questionnaire that classifies subjects as non-stressed or stressed and, in the latter case, defines the phase of stress (alarm, resistance, pre-exhaustion and exhaustion). Only caregivers whose scores were associated with chronic stress (resistance, pre-exhaustion and exhaustion phases) and non-caregivers who were classified as non-stressed were included in the study. The body mass index (BMI) of all participants was evaluated.

This study was approved by the Research Ethics Committee of the Pontifical Catholic University of Rio Grande do Sul (Porto Alegre, Brazil) and was therefore performed in accordance with the ethical standards of the 1964 Declaration of Helsinki. All participants gave their written informed consent.

### 2.2 Neuropsychological Measures

Frontal lobe functions were assessed with neuropsychological tests that measured different components of executive function. The Digit-span tests [34] were employed to assess working memory. Trail Making A and B tests and the word (I) and color (II) versions of the Stroop test were used to evaluate attention and processing speed [35]. The word/color (III) version of the Stroop test was used to evaluate the inhibitory response capacity [35].

Temporal lobe functions were assessed by the Logical Memory Test [36]. This task evaluates immediate and delayed recall of declarative memory and is heavily dependent on hippocampal formation.

All procedures related to the neuropsychological assessments followed the recommended guidelines for each specific task and have been briefly described elsewhere [19].

### 2.3 Cortisol and DHEA Analysis

As previously described [19], participants were asked to collect saliva samples at home at 8 AM and 10 PM on the day of the neuropsychological assessment. The samples were stored between 0°C and 4°C by the subjects and were delivered to the laboratory within 3 days, where they were frozen at −80°C until further analysis. After thawing, each sample was divided for cortisol and DHEA assessment. Samples for cortisol analysis were centrifuged at 1500 rpm for 3 min and then analyzed via a radioimmunoassay (Beckman Coulter kit, Immunotech) using a gamma counter. The assay sensitivity was estimated at 0.09 nmol/L. Samples for the DHEA analysis were centrifuged for 3 min at 2500 rpm and then measured by radioimmunoassay (Beckman Coulter kit, Immunotech). The sensitivity of the DHEA assay was estimated at 0.06 nmol/L. All samples for both cortisol and DHEA were analyzed in duplicate, and the results from each of the sampling times were expressed in nmol/L [37].

### 2.4 BDNF

A nursing professional collected 5 ml of peripheral blood from each volunteer via venipuncture into an anticoagulant-free vacuum tube. The clotted blood samples were then centrifuged at 4000 rpm for 10 minutes, and the serum was kept frozen at -80°C until further analysis. As previously described [38], the serum BDNF analysis was performed using an ELISA kit following the manufacturer’s instructions (Millipore, USA). In short, microtiter plates (96-well flat-bottom) were coated for 24 h at 4°C with the samples diluted 1:100 in sample diluent and the standard curve ranging from 7.8 to 500 ng/ml of BNDF. The plates were then washed four times with wash buffer followed by the addition of biotinylated mouse anti-human BNDF monoclonal antibody (diluted 1:1000 in sample diluent), which was incubated for 3 h at room temperature. After washing, a second incubation was carried out with streptavidin-horseradish peroxidase conjugate solution (diluted 1:1000) for 1 h at room temperature. After addition of the substrate and stop solution, the amount of BDNF was determined (absorbance set at 450 nm). The standard curve demonstrates a direct relationship between optical density and BDNF concentration [38].

### 2.5 Statistical Analysis

Demographic and clinical characteristics were analyzed using chi-squared statistics, a one-way analysis of variance (ANOVA) and independent samples t tests, where appropriate. Cognitive performance and BDNF levels were submitted to two-way ANOVAs, with age (younger X older subjects) and chronic stress (caregivers X controls) as the between-group variables. These two-way ANOVAs were followed by one-way ANOVAs and Bonferroni’s post hoc test, when necessary. The cortisol, DHEA and cortisol/DHEA levels were analyzed with a mixed design analysis of variance (MANOVA), with age (younger X older subjects) and chronic stress (caregivers X controls) as the between-group variables and the sampling time (8AM and 10PM) as the within group variables. These MANOVAs were also followed by ANOVAs and Bonferroni’s post hoc test. To strengthen the internal validity and generalizability of our results, we also ran a one-way analysis of covariance (ANCOVA) for all variables (neuropsychological tests, BDNF and hormonal levels). In addition to BAI and BDI scores, which showed significant between-group differences, we also included gender, education, MMSE and BMI in the covariance analysis, even in the absence of significant between-group differences (see the results for demographic and clinical characteristics below). The rationale for doing so came from the literature, as these variables routinely affect the outcomes of neuropsychological, cortisol, DHEA and BDNF data [27,39–46]. Finally, linear regressions were run between the results of neuropsychological tests and hormonal and BDNF levels. The results are expressed as the means ± standard error. The statistical significance was set at P < 0.05. The power of all statistical analysis was greater than 80% and the effect sizes [eta squared (ƞ2ƿ) or Rsquare (R2)] are reported for all statistically significant results.

## 3 Results

### 3.1 Demographic and Clinical Characteristics

Significant age differences [ƞ2ƿ = 0.749, p<0.001] were observed between younger and older subjects (p<0.001) but not between the two younger (p = 1.00) or the two older groups (p = 0.094). There were also no significant differences between groups for gender [Pearson Chi-Square = 1.582, p = 0.663], years of education [p = 0.232], MMSE [p = 0.5] or BMI [p = 0.852]. On the other hand, scores of the depressive [ƞ2ƿ = 0.613, p<0.001] and anxiety [ƞ2ƿ = 0.441, p<0.001] screening tests were significantly different between groups. As shown by Bonferroni’s post hoc test, the scores of younger and older caregivers on BDI and BAI were similar (all p>0.05) and higher than the scores of the control groups (all p< 0.01) (Table 1). There was also no significant difference between the caregiver groups for the time devoted to patient assistance (hours/week and years) or the prevalence of physical and psychological stress symptoms (all p>0.05).

**Table 1:** Demographic and psychiatric measures (mean ± standard error) of the different age groups of controls and caregivers.

	Younger Controls	Younger Caregivers	Older Controls	Older Caregivers
Age (years)	46.23 ± 1.37	48.82 ± 2.07	68.22 ± 1.51 *	74.16 ± 1.82 *
Sex (F/M)	14/3	15/2	13/5	15/3
Education (years)	13.52 ± 0.53	13.58 ± 0.52	11.88 ± 0.84	12.94 ± 0.62
MMSE	28.05 ± 0.29	28.35 ± 0.34	27.38 ± 0.43	27.05 ± 0.33
BDI	5.70 ± 0.78	15.70 ± 1.30 **	4.27 ± 0.88	16.61 ± 1.31 **
BAI	3.05 ± 0.58	7.29 ± 0.89 **	2.94 ± 0.80	10.61 ± 1.16 **
BMI	24.80 ± 1.16	24.96 ± 1.03	25.93 ± 0.97	24.70 ± 0.97
Assisted Time (Weekly Hours)		99.76 ± 14.82		136.88 ± 12.63
Caregiving (years)		3.52 ± 0.44		5.27 ± 0.87
Symptoms of Stress (ISSL) n (%)				
Physical		5 (29)		4 (22)
Psychological		12 (71)		14 (78)

Abbreviations: MMSE, Mini Mental Status Examination; BDI, Beck Depression Inventory; BAI, Beck Anxiety Inventory and BMI, Body Mass Index; ISSL, Lipp Stress Symptoms Inventory for Adults.

* p<0.05 compared to younger controls and caregivers

** p<0.01 compared to controls

Some caregivers (30.7% of the younger sample and 38.4% of the older sample) were taking antidepressant and/or anxiolytic medications: six volunteers were taking only antidepressants [selective serotonin reuptake inhibitor (SSRI), n = 4; monoamine oxidase inhibitor (MAOI), n = 1; tricyclic plus SSRI, n = 1]; two used antidepressants in combination with anxiolytic medication [SSRI plus benzodiazepinic, n = 2] and another used only anxiolytic medication [bupropion, n = 1]. An exploratory statistical analysis with independent samples t tests indicated no significant differences (all p>0.05) in the levels of depressive and anxiety symptoms, hormones, BDNF and neuropsychological scores between the medicated and unmedicated caregivers of the younger and older samples and thus we were confident in including the medicated caregivers in the sample.

### 3.2 Neuropsychological Data

The two-way ANOVAs indicated significant age effects on working memory [ƞ2ƿ = 0.188, p<0.001 for Forward Digit span; ƞ2ƿ = 0.080, p = 0.019 for Backward Digit span], attention and processing speed [ƞ2ƿ = 0.253, p<0.001 for Trail Making A, ƞ2ƿ = 0.247, p<0.001 for Trail Making B, ƞ2ƿ = 0.340, p<0.001 for Stroop I and ƞ2ƿ = 0.458, p<0.001 for Stroop II] and inhibitory response capacity [ƞ2ƿ = 0.376, p<0.001]. Further investigations of these results using ANOVAs [ƞ2ƿ = 0.397 to 0.645, all p<0.05] and Bonferroni’s post hoc tests showed that older controls had lower performances than younger controls for all tasks (all p<0.01), with the exception of Trail Making A [p = 1.00], for which an interaction between age and stress was observed in the two-way ANOVA [ƞ2ƿ = 0.125, p = 0.003], thus limiting the age effect to older caregivers. Moreover, older caregivers also showed significantly worse performances than their younger counterparts in all tasks cited above [p<0.001], with the exception of the Backward Digit span [p = 0.754] (Table 2).

**Table 2:** Performances (mean ± standard error) of younger and older controls and caregivers on neuropsychological tests.

	Younger Controls	Younger Caregivers	Older Controls	Older Caregivers
Digit Span (Forward)	7,17 ± 0.26	5.11 ± 0.22 #	6.44 ± 0.33	3.88 ± 0.13 *
Digit Span (Backward)	5.88 ± 0.18	2.88 ± 0.26 #	5.16 ± 0.39	2.27 ± 0.17 #
Trail Making A	34.88 ± 1.97	39.94 ± 2.77	40.22 ± 2.68	65.11 ± 4.66 *
Trail Making B	67.05 ± 3.29	102.17 ± 6.75 ¤	102.27 ± 13.80 ¤	144.44 ± 4.53 *
Stropp I	93.17 ± 2.14	77.29 ± 2.68 ¤	75.27 ± 3.87 ¤	62.22 ± 2.15 *
Stropp II	73.64 ± 2.09	59.47 ± 1.94 ¤	56.00 ± 2.73 ¤	43.11 ± 2.19 *
Stropp III	54.70 ± 2.10	37.05 ± 2.14 ¤	35.38 ± 3.40 ¤	24.83 ± 2.00 *
Logic Memory I	27.70 ± 0.63	18.52 ± 1.35 #	26.50 ± 1.02	17.50 ± 0.87 #
Logic Memory II	23.52 ± 1.00	13.70 ± 0.99 #	20.61 ± 1.11	13,33 ± 0.86 #
				

* p<0.05 compared to all groups

# p<0.05 compared to control groups

¤ p<0.05 compared to younger controls group

The statistical analysis also showed significant effects of chronic stress on all cognitive functions investigated, as observed for the results of the two-way ANOVAs for working memory [ƞ2ƿ = 0.562, p<0.001 for Forward Digit span; ƞ2ƿ = 0.634, p< 0.001 for Backward Digit span], attention and processing speed [ƞ2ƿ = 0.246, p<0.001 for Trail Making A; ƞ2ƿ = 0.246, p<0.001 for Trail Making B; ƞ2ƿ = 0.284, p<0.001 for Stroop I and ƞ2ƿ = 0.349, p<0.001 for Stroop II], inhibitory response capacity [ƞ2ƿ = 325, p<0.001] and declarative memory [ƞ2ƿ = 0.554, p<0.001 for Logical Memory I; ƞ2ƿ = 0.525, p<0.001 for Logical Memory II]. Further analyses of these results using ANOVAs [ƞ2ƿ = 0.397 to 0.645, all p<0.05] and Bonferroni’s post hoc tests confirmed that younger and older caregivers had significantly lower scores than their age-matched controls for all neuropsychological tasks [all p<0.05]. The only exception was in Trail making A. As discussed above, this task showed and interaction between age and stress [ƞ2ƿ = 0.125, p = 0.003], limiting the stress effect to older caregivers. It is also important to draw attention to the fact that the performance of younger caregivers was significantly lower that of older controls in the Forward and Backward Digit Span and in the Logical Memory I and II tests (all p<0.05). On the other cognitive tasks (Trail Making A and B, and Stroop I, II and III), no significant differences were found between younger caregivers and older healthy controls (Table 2).

To summarize, our results indicate that chronic stress due to caregiving activities (I) usually promoted greater deficits in older caregivers than in younger caregivers (working memory, processing speed and inhibitory control) and (II) impaired younger caregivers in such manner that their performance fell to the same (attention, processing speed, inhibitory control) or even lower (working and declarative memory) levels than the older controls.

### 3.3 Hormonal Levels

**Fig 1:**
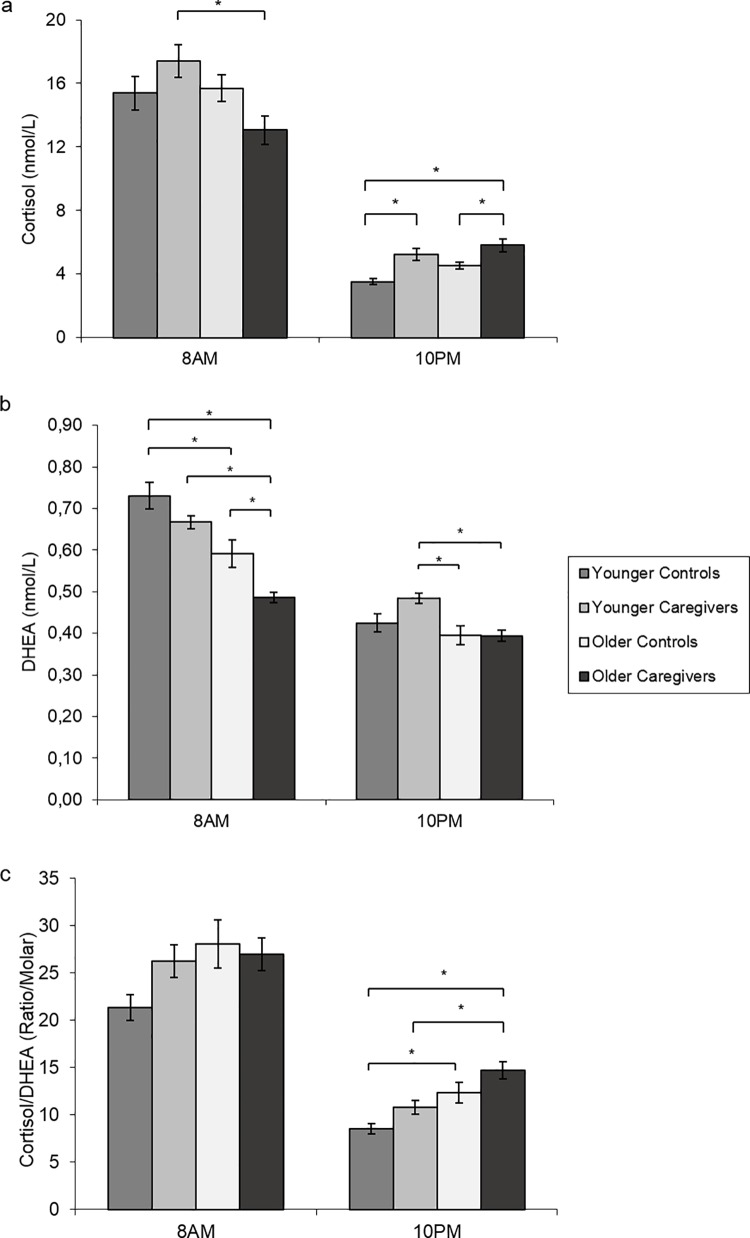
Age and/or stress effects at 8AM and 10PM based on the saliva levels of cortisol (a), DHEA (b) and the cortisol/DHEA ratio (c). The results expressed as the means ± standard error. * p < 0.05.

Fig 1 shows the levels of cortisol (1a) and DHEA (1b) and the ratio of these hormones (1c).

#### 3.3.1 Cortisol levels

The mixed ANOVA indicated a significant effect of time on cortisol levels [ƞ2ƿ = 0.902, p<0.001] and an interaction among time, age and stress [ƞ2ƿ = 0.084, p = 0.016]. The significant time effect can be explained by the higher cortisol levels at 8 AM than at 10PM in all experimental groups (all p<0.001). The interaction between time, age and stress can be better appreciated by the analysis of the group differences, as indicated by the ANOVAs at 8AM [ƞ2ƿ = 0.137, p = 0.021] and 10PM [ƞ2ƿ = 0.293, p<0.001]. Bonferroni’s post hoc test showed an age effect at 8AM among the stressed subjects, such that cortisol levels were higher for younger caregivers than for older caregivers (p = 0.003). No significant differences were observed for the 8AM levels of this steroid between controls and caregivers (p>0.05), nor between younger and older controls (p>0.05). At 10PM, a different pattern of results emerged: the cortisol levels of the younger and older caregivers were similar (p>0.05) and were higher than the levels of their respective age-matched controls (all p<0.05). In short, chronic stress effects on the cortisol levels of younger and older caregivers were observed only at 10 PM.

#### 3.3.2 DHEA levels

The MANOVA of DHEA results indicated a significant effect of time [ƞ2ƿ = 0.824, p<0.001] and age [ƞ2ƿ = 0.345, p<0.001], but no effect of stress was detected [ƞ2ƿ = 0.032, p = 0.145]. However, there was also a significant interaction between time and stress [ƞ2ƿ = 0.285, p = 0.001]. Further analysis of these results with ANOVAs indicated significant between-group differences at 8AM [ƞ2ƿ = 0.450, p<0.001] and at 10PM [ƞ2ƿ = 0.198, p = 0.002]. Bonferroni’s post hoc tests indicated a significant decline in DHEA levels with age for controls (p<0.001) and caregivers (p<0.001) at 8AM. Older caregivers had the lowest DHEA levels of all groups at this sampling time (all p<0.05), whereas younger caregivers showed similar levels of this hormone as their respective age-matched control group (p = 0.490). At 10PM, no significant age differences were observed between the younger and older controls (p>0.05). However, younger caregivers showed higher DHEA levels than older controls (p = 0.005) and caregivers (p = 0.005). To summarize, a clear stress effect on DHEA levels was observed only at 8AM and only for older caregivers.

#### 3.3.3 Cortisol/DHEA ratios

The results obtained with the mixed ANOVA for cortisol/DHEA ratios indicated a significant effect of time [ƞ2ƿ = 0.842, p<0.001] and age [ƞ2ƿ = 0.116, p = 0.004], no effect of stress [ƞ2ƿ = 0.040, p = 0.102] and a significant interaction among time, age and stress [ƞ2ƿ = 0.061, p = 0.043]. The significant time effects can be explained by the higher cortisol/DHEA ratios at 8AM than at 10PM in all experimental groups (all p<0.05). The one-way ANOVAs indicated significant group differences only at 10 PM [ƞ2ƿ = 0.298, p<0.001]. At this sampling time, we can see a clear age effect, with younger controls and caregivers showing lower cortisol/DHEA ratios then their respective older counterparts (all p<0.05). The time, age and stress interaction can be understood when we realize that older caregivers had the highest levels (p<0.01 in relation to younger subjects) at 10PM. In short, the cortisol/DHEA ratios suggest interactions between age and stress only at 10PM; therefore, older caregivers seem to be the most affected by these variables.

### 3.4 BDNF Levels

**Fig 2:**
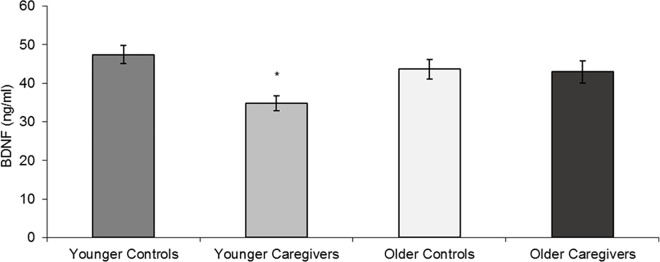
BDNF levels (mean ± standard error) of younger and older controls and caregivers. * p < 0.05 compared to younger controls.

The two-way ANOVA of BDNF levels showed a clear effect of stress [ƞ2ƿ = 0.097, p = 0.010] and an interaction between stress and age [ƞ2ƿ = 0.079, p = 0.021] on the levels of this neurotrophin. However, age alone had no effect on BDNF levels [ƞ2ƿ = 0.011, p = 0.390]. ANOVA and Bonferroni’s post-hoc tests indicated that the significant group differences [ƞ2ƿ = 0.165, p = 0.007] observed for BDNF were among younger caregivers and their corresponding age-matched controls (p = 0.005), as shown in Fig 2. Thus, stress and age interacted to lower the levels of this neurotrophin in younger caregivers.

### 3.5. Covariance Analysis for Neuropsychological Performance, BDNF and Hormonal Levels

As stated earlier, to strengthen the internal validity and generalizability of the results presented above, separate ANCOVAs were run to evaluate the effect of each covariate (age, gender, education, BMI, BAI scores and BDI scores) on each neuropsychological task, on BDNF levels and on cortisol, DHEA and cortisol/DHEA ratios at each sampling time (8AM and 10PM). These ANCOVAs indicated that the demographic (gender, education and BDI) and clinical (MMSE, BAI and BDI scores) characteristics of our samples had no significant effects (all p>0.05) as covariates and, consequently, did not alter the significant group differences described earlier for the neuropsychological, hormonal and BDNF outcomes.

### 3.6 Relations among Cognition and Hormonal or BDNF Levels

Separate linear regressions were run for younger and older subjects to search for relationships between cognitive performance (dependent variable) and physiologic parameters that were significantly altered by stress (hormonal and BDNF levels, independent variables) within the different age groups.

The results of these linear regressions indicated that younger subjects had a significant relationship between scores on neuropsychological tests and cortisol levels only for the Trail Making B task [R2 = 0.316, B = 10,393, p = 0.001]. None of the linear regressions evaluated for older subjects showed any significant relationship between cognitive performance and cortisol levels (all p>0.05). On the other hand, DHEA levels of older subjects showed significant relationships with performance on the Forward and Backward Digit Span and on the Trail Making B and Logical Memory I and II tasks (all p<0.05) (Table 3).

**Table 3:** Results of the linear regressions for cognitive performance and the physiological parameters (DHEA or BDNF) in younger and older subjects.

	**Older subjects DHEA at 8AM**		
	R2	B	*p*
Span Forward	0.187	6,319	0.008
Span Reverse	0.265	8,712	0.001
Trail Making B	0.150	-161,086	0.020
Logical Memory	0.151	20,419	0.019
Logical Memory	0.123	16,953	0.036
	**Younger subjects BDNF**		
	R2	B	*p*
Span Forward	0.377	0.080	<0.001
Span Reverse	0.475	0.113	<0.001
Stroop I	0.233	0.561	0.004
Stroop II	0.139	0.371	0.030
Stroop III	0.145	0.432	0.0026
Logical Memory I	0.180	0.245	0.013
Logical Memory II	0.327	0.335	<0.001

The BDNF levels of younger subjects were significantly related to most of the analyzed cognitive domains, including the working memory [Forward (p<0.001) and Backward Digit Span (p<0.001)], attention [Stroop I (p = 0.004) and II (p = 0.030)], inhibitory response [Stroop III (p = 0.026)] and memory [Logical Memory I (p = 0.013) and II (p<0.001)] regressions.

In general, the results described above revealed that (I) cortisol levels at 10PM were not related to the cognitive outcomes of younger or older subjects, with the exception of Trail B performance in younger volunteers; (II) decreased levels of DHEA at 8AM were related to the worst cognitive outcome in older subjects; and (III) lower BDNF levels were related to a decrease in the cognitive performance of younger subjects.

## 4 Discussion

The aim of this study was to investigate the impact of chronic stress due to caregiving of AD patients on cognition, hormonal and BDNF levels of younger and older familial caregivers. The results indicated cognitive impairments in both caregiver groups, with surprisingly important deficits in younger caregivers, for which their performance fell to the same or lower levels as healthy older controls, suggesting a precocious cognitive aging. Moreover, cortisol levels at 10PM were increased in both caregiver groups, whereas DHEA levels at 8AM fell only among the older caregivers. These hormonal alterations were not able to induce significant differences on cortisol/DHEA ratios between caregivers and their respective age controls. Even so, lower DHEA levels at 8AM were significantly related to the worst cognitive outcome in older subjects. On the other hand, BDNF levels showed a decrease only in younger caregivers, which was related to a decrease in cognitive performance.

A significant emotional burden, characterized by the prevalence of psychological stress symptoms on the ISSL scale, was observed among younger and older caregivers, presumably as a consequence of the long lasting, high weekly load of caregiving activities and the emotional suffering due to the close relationship between caregivers and patients [5,47]. In accordance with this scenario, we also found more depression and anxiety symptoms among caregivers. Nevertheless, the BDI and BAI scores for both caregiver groups were below the cutoff for moderate depression and anxiety symptomatology [40], and this likely explains the lack of an observed effect of the scores of BAI and BDI as significant covariates in the neuropsychological, hormonal and BDNF analyses. The depression and anxiety symptoms were likely maintained at low levels because some caregivers were taking antidepressant and/or anxiolytic medication, as described in the results section. As shown in previous studies, the use of such medications (and the presence of depression and anxiety-related disorders) is very common among caregivers [47,48].

The cognitive impairments observed in this study for older caregivers’ working and declarative memory, attention, processing speed and inhibitory response capacity are in accordance with many other studies [3,7,8,49–52]. However, our results add two new important pieces of information. First, older caregivers are especially prone to the negative effects of caregiving activities on prefrontal functions, as suggested by the greater cognitive decline in prefrontal-dependent tasks than in younger caregivers. Until now, this result was only hypothesized in the literature [3,52], based on the knowledge that both age and chronic stress are able to predispose individuals to cognitive decline [53]. Second, younger caregivers seem to be predisposed to a precocious cognitive aging. In addition to the lower performance of younger caretakers than of younger controls in nearly all neuropsychological tests, the scores of the younger caretakers on the prefrontal and temporal lobe-dependent tasks decreased to the same (attention and processing speed, inhibitory control and declarative memory) or lower (working memory) levels of older controls. It is important to highlight that some studies have reported increased incidence rates for dementia among the spouses of persons with dementia [3,7]. However, these studies focused on caregiving activities as late-life stressors. The expected dementia incidence rate among middle-aged familial caregivers, such as AD patients’ children, for which this psychosocial stressor is initiated earlier has not been reported. The results of the neuropsychological tests of our younger caregivers clearly point to their risk of cognitive decline and the need of more studies on this age group.

In contrast to most other chronic stress studies, we analyzed cortisol secretion only at 8AM and 10PM. Although not common, this method was chosen because it allowed caregivers to collect saliva samples at home (increasing study adherence), without the complications associated with multiple samplings throughout the day [54] or the risk of compromising the strict standardization and timing required by other techniques, such as the cortisol awaking response [12,55]. Our experimental paradigm showed that caregivers had a typical rhythm of cortisol secretion [56,57], with serum concentrations decreasing form the morning to the night. However, the levels of this hormone at 10PM were clearly influenced by stress, showing higher values for younger and older caregivers than for their respective age-matched controls. Similar results were found in previous studies by our group and other research groups [19; 13; 51] and could be explained by an HPA imbalance [19; 52] and/or behavioral alterations of patients at early night, known as Sundowning Syndrome, which can impose extra difficulties (and stress) for caregivers [58].

A clear stress effect on DHEA was observed only at 8AM and only for older caregivers. Similar DHEA alterations have also been reported by other studies on caregivers [59], although controversies exist [19]. It is worth noting that our elderly caregivers also had lower mean levels of cortisol than their age-matched controls at 8AM (although statistical significance was not reached), probably reflecting the expected positive, albeit weak, correlation between cortisol and DHEA levels in response to HPA axis control [60].

As expected, cortisol/DHEA ratios tended to increase in caregivers, but statistical significance was not reached. Although the cortisol/DHEA ratio seems to be a more reliable marker for cognitive changes than cortisol or DHEA alone [21], only two other studies investigated this ratio in caregivers. Corrêa and colleagues [19] found increased cortisol/DHEA ratios at 8AM and 10PM, mainly due to increases in cortisol levels. Jeckel and collaborators [59] also found increased cortisol/DHEA ratios in caregivers; however, their results were the consequence of low DHEA levels, not increasing cortisol values.

The discrepancies observed among different studies on caregivers’ cortisol, DHEA and cortisol/DHEA levels could be explained by the different age compositions of the experimental samples, as suggested by the age effects observed in this and other studies [10]. Moreover, other factors, such as caregivers’ stress levels, coping strategies [61], and the stage of the patient’s disease [62], could contribute variability to the data. Thus, it is important to emphasize the need for more studies to adequately investigate each of these parameters and their contribution to hormonal alterations in caregivers.

In addition to cortisol and DHEA levels, we also investigated the serum BDNF of caregivers. Previous studies found that chronic stress can influence neurotrophin levels [26]. Researchers suggest a possible relation between hypercortisolemia and lower BDNF levels [26,63,64], and recently, our laboratory reported, for the first time, a decrease in BDNF levels in the familial caregivers (32–84 years old) of AD patients [19]. In the present study, we also found decreased levels of BDNF in caregivers, but only for the younger caregivers. Thus, our results point to an interaction of age and chronic stress on BDNF levels such that younger caregivers are more affected than the older caregivers.

Assuming that peripheral cortisol, DHEA and BDNF are related to their respective brain levels [65,66] and because they are involved in cellular and molecular mechanisms of cognition [26], we investigated whether the effects of chronic stress on these physiologic parameters were related to the cognitive impairment observed in caregivers. Linear regressions between these variables revealed that cortisol levels at 10PM were not related to the cognitive outcomes of younger or older subjects. However, decreased levels of DHEA at 8 AM were related to the worst cognitive outcome in older subjects, while lower BDNF levels were related to a decrease in the cognitive performance of younger subjects. More specifically, decreased DHEA levels were related to worsening outcomes on working memory, processing speed and declarative memory (R² ranging from 0.12–0.27), suggesting that DHEA could be one of the mediators of the effects of chronic stress on cognitive impairments in older caregivers [21]. In turn, BDNF levels were correlated with all cognitive domains assessed in the younger subjects (R² ranging from 0.13–0.47), such as working memory, attention, inhibitory response capacity, processing speed and declarative memory.

Based on the discussion above, it is clear that the sample size was a limitation of the current study. Although our results on cognitive performance were in agreement with our hypothesis and previous reports in the literature, a larger sample may have been more accommodating for drawing stronger conclusions regarding hormonal levels, cortisol/DHEA ratios and BDNF levels and regarding the relationships among these parameters with cognition. Moreover, some of our caregivers were using medication for depression and anxiety, suggesting that they suffer from depression and/or anxiety-related disorders. Thus, an impact of their condition or of the medication they were taking on the variables analyzed in this study cannot be ruled out. Even so, it seems that these aspects did not affect our results because no significant differences were identified in the levels of depressive and anxiety symptoms, hormones, BDNF and neuropsychological scores between the medicated and unmedicated caregivers. Moreover, caregivers used different medications, with opposing effects reported for the investigated parameters (cognition, hormonal and BDNF levels), depending on their dosage, usage time and combination [67–69]. Thus, it seems very unlikely that these medications deviated the obtained results in a specific direction and altered our conclusions. Nevertheless, it would be wise to design future studies to investigate the potential effects of antidepressant and/or anxiolytic medications and the effect of depression and anxiety-related disorders on caregiver’s outcomes.

## 5 Conclusions

The present study showed that younger caregivers exhibited significant cognitive dysfunctions and that the cognitive performance of older caregivers is even more compromised. Hormonal and BDNF levels were affected by the chronic stress of caregivers and were partially related to their cognitive impairments. We are confident that our results expand the knowledge in this area, and we hope that our results draw the attention of policymakers and clinicians to the fact that the mental health of middle-aged caregivers deserves as much attention as the mental health of older caregivers. Even minor cognitive problems in caregivers may affect their quality of life and their ability to provide adequate care, which represent major implications for formal health systems at a time when demand is increasing. Thus, the development of interventions aimed to help families with AD patients to manage the stressful effects of caregiving activities is urgent.
